# Human Gut Antibiotic Resistome and Progression of Diabetes

**DOI:** 10.1002/advs.202104965

**Published:** 2022-02-10

**Authors:** Menglei Shuai, Guoqing Zhang, Fang‐fang Zeng, Yuanqing Fu, Xinxiu Liang, Ling Yuan, Fengzhe Xu, Wanglong Gou, Zelei Miao, Zengliang Jiang, Jia‐ting Wang, Lai‐bao Zhuo, Yu‐ming Chen, Feng Ju, Ju‐Sheng Zheng

**Affiliations:** ^1^ Key Laboratory of Growth Regulation and Translational Research of Zhejiang Province School of Life Sciences Westlake University Hangzhou 310030 China; ^2^ Westlake Intelligent Biomarker Discovery Lab Westlake Laboratory of Life Sciences and Biomedicine Hangzhou 310024 China; ^3^ Key Laboratory of Coastal Environment and Resources of Zhejiang Province School of Engineering Westlake University Hangzhou 310030 China; ^4^ Institute of Advanced Technology Westlake Institute for Advanced Study Hangzhou 310024 China; ^5^ Guangdong Provincial Key Laboratory of Food Nutrition and Health Department of Epidemiology School of Public Health Sun Yat‐sen University Guangzhou 510275 China; ^6^ Department of Epidemiology School of Medicine Jinan University Guangzhou 510632 China; ^7^ Institute of Basic Medical Sciences Westlake Institute for Advanced Study Hangzhou 310024 China

**Keywords:** cardiometabolic traits, diabetes, fecal metabolome, human gut antibiotic resistome, metagenomics, population‐based cohort study

## Abstract

The antibiotic resistance crisis underlies globally increasing failures in treating deadly bacterial infections, largely due to the selection of antibiotic resistance genes (ARG) collection, known as the resistome, in human gut microbiota. So far, little is known about the relationship between gut antibiotic resistome and host metabolic disorders such as type 2 diabetes (T2D). Here, metagenomic landscape of gut antibiotic resistome is profiled in a large multiomics human cohort (*n* = 1210). There is a significant overall shift in gut antibiotic resistome structure among healthy, prediabetes, and T2D groups. It is found that larger ARG diversity is associated with a higher risk of T2D. The novel diabetes ARG score is positively associated with glycemic traits. Longitudinal validation analysis confirms that the ARG score is associated with T2D progression, characterized by the change of insulin resistance. Collectively, the data describe the profiles of gut antibiotic resistome and support its close relationship with T2D progression.

## Graphical abstract

Little is known about the relationship between gut antibiotic resistance genes (ARG) and diabetes. This study profiles the gut antibiotic resistome in a large human cohort (*n*=1210). There is a significant overall shift in the ARG composition across healthy, prediabetes, and diabetes individuals. A novel diabetes‐ARG score is associated with diabetes progression, characterized by the alteration of insulin resistance.

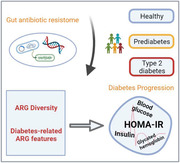

## 1 Introduction

The global rise of antibiotic resistance jeopardizes the success and sustainability of modern medication to fight against deadly multidrug resistant infections.^[^ ^1^ ^]^ Human gut is the key reservoir of microbiota harboring both commensal and pathogenic bacteria.^[^ ^2^ ^]^ It, however, also represents a hotspot for the frequent selection of diverse antibiotic resistance genes (ARGs) and their bacterial hosts due to the regular exposure of gut microbiota to foreign antibiotics directly from human medications and indirectly from food chain.^[^ ^3^ ^]^ While current cohort studies have largely focused on the microbiome diversity and its association with human disease and health, the research on gut antibiotic resistome, i.e., the collection of ARGs within microbiome, is rare in large human cohorts. The antibiotic exposure usually leads to long‐term enrichment of ARGs in gut microorganisms,^[^ ^4^ ^]^ but its consequence and relation with human metabolic health were not clear.

We generated a hypothesis that gut antibiotic resistome may be associated with type 2 diabetes (T2D) progression, given that prior evidence suggested a close link between human gut microbiome and T2D,^[^ ^5^ ^]^ and that self‐reported long‐term use of antibiotics was associated with higher T2D risk and future cardiovascular diseases.^[^ ^6^ ^]^ Moreover, due to the genetic and lifestyle heterogeneities across different populations, we argued that compared with the prescriptions and questionnaires for inquiring antibiotic use, tracking the antibiotic resistome of gut microbiome would be more informative and help provide more mechanistic insight.

Therefore, the aim of the present study was to depict the profiles of gut antibiotic resistome in a large human cohort and explore its relationship with gut microbial metabolites and host metabolic health, which is a key step to understand the influence of the gut antibiotic resistome on human health.

## 2 Results

### 2.1 Composition and Variation of the Gut Antibiotic Resistome

**Figure 1:**
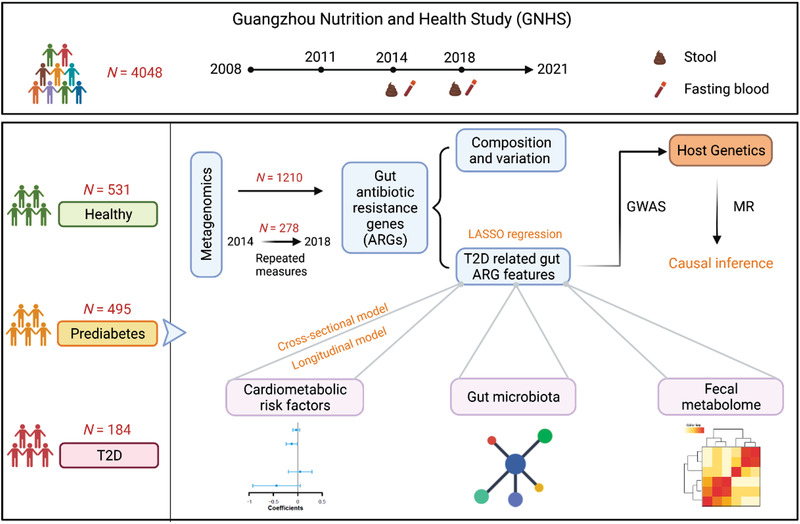
Study strategy for linking gut antibiotic resistome and progression of T2D. Our study was based on the Guangzhou Nutrition and Health Study (GNHS) which is an ongoing community‐based cohort study in China. We included 1210 participants in the current study, of whom 531 were healthy, 495 were prediabetes and 184 were T2D. To associate the gut antibiotic resistome and T2D, we profiled the stool metagenome. 278 individuals have collected stool sample twice at different time points. We applied ARG‐OAP2 and MetaPhlAn2 to perform the profiling of the gut antibiotic resistome and gut microbiota, respectively. To explore the potential causal association of the identified ARG features with T2D, we performed genome‐wide association analyses (GWAS) and one‐sample Mendelian randomization (MR) analyses. In addition, we examined the associations between gut ARG features and cardiometabolic traits using the cross‐sectional model, which was validated by the longitudinal model. We also constructed the co‐occurrence network between gut ARGs and microbial species. Finally, Spearman correlation analysis was used to investigate the associations between T2D related ARG features and fecal metabolome.

We included 1210 participants with a mean age of 64.9 years (SD: 5.5) and with fecal metagenomics data from the GNHS into our present analysis (**Figure** **1**).^[^ ^7^ ^]^ We assessed the diabetes status of the participants, of whom 531 were healthy, 495 were prediabetes and 184 were T2D (Table S1, Supporting Information). Using paired‐end shotgun metagenomic sequencing, we obtained 42.4 million paired‐end reads on average (Table S2, Supporting Information).

**Figure 2:**
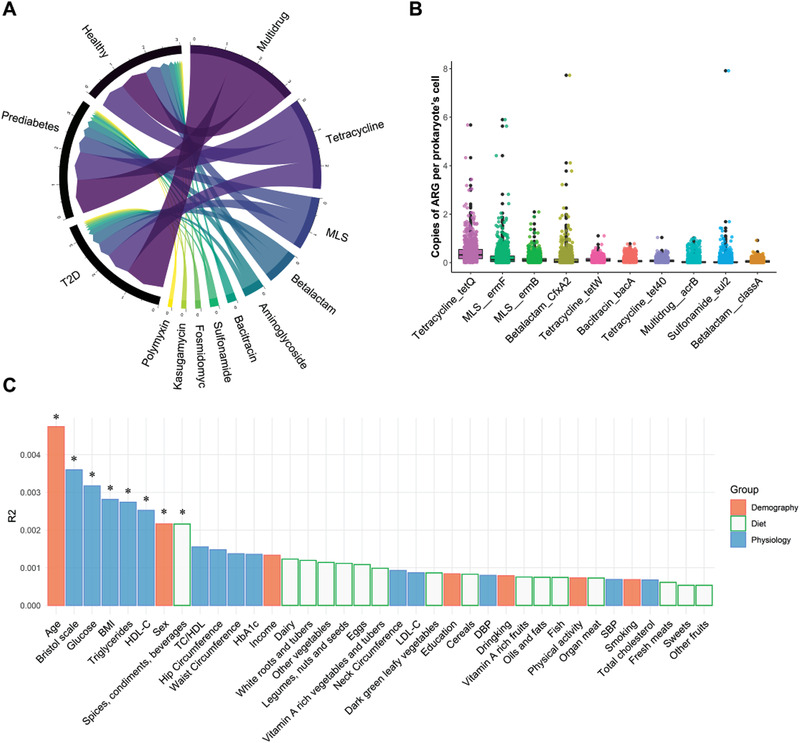
Compositional variation in human gut antibiotic resistome. A) Circus plot showing the mean relative abundance of top 10 antibiotic resistance genes (ARGs) types among Healthy (*n* = 531), Prediabetes (*n* = 495) and Type 2 Diabetes (T2D, *n* = 184) groups. B) Box plot showing the abundance of top 10 ARGs subtypes (*n* = 1210). C) The effect sizes of host factors on human gut antibiotic resistome were calculated by PERMANOVA (Adonis, permutations = 999) (*n* = 947). *MLS, Macrolide‐Lincosamide‐Streptogramin*.

We detected a total of 19 ARG types and 805 ARG subtypes (Figure S2, Supporting Information). The most abundant ARG types in this study included *Multidrug*, *Tetracycline* and *Macrolide‐Lincosamide‐Streptogramin (MLS)*, followed by *Beta‐lactam*, *Aminoglycoside* and *Bacitracin* (**Figure** **2**A). After 10% prevalence filtering, 17 ARG types and 233 subtypes remained for further analysis. We observed that *Tetracycline*_*TetQ* was the most abundant ARG subtype in our participants (Figure 2B). The prevalence of *Quinolone*, *Quinolone_norB* and subtypes of *Beta‐lactam* increased along with the diabetes progression (Figure S4A–C, Supporting Information). Overall, 17 core ARG subtypes were shared by all of the participants among healthy, prediabetes, and T2D groups (Figure S4D, Supporting Information).

We correlated 7 demographic factors, 16 dietary intakes and 14 physiological factors to the inter‐individual variation in the gut antibiotic resistome. Age had the largest explanatory power on the gut antibiotic resistome compositional difference (Bray‐Curtis distance, PERMANOVA, *p* = 0.002), which may be due to the accumulation of ARGs during the lifespan and become more diversified with age.^[^ ^8^ ^]^ A total of 8 factors were found to be significantly associated with the overall resistome variation (Figure 2C; Table S3, Supporting Information). In addition, we repeated our analysis among healthy, prediabetes and T2D groups, respectively. Our data showed different patterns for the contribution of those factors, which indicates the impact of diabetes status on the relationship between gut antibiotic resistome and those factors (Figure S5, Supporting Information).

### 2.2 Gut Antibiotic Resistome Composition are Associated with Type 2 Diabetes

**Figure 3:**
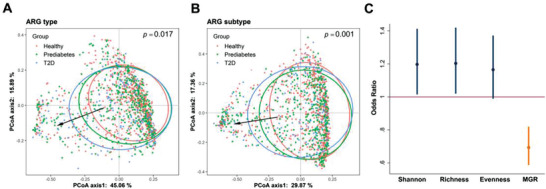
Gut antibiotic resistance genes are associated with type 2 diabetes. A,B) PCoA plots showing the compositional differences of gut antibiotic resistome among different diabetes groups. *p* was calculated by PERMANOVA (Adonis, permutations = 999) based on Bray‐Curtis distance. C) Forest plot showing the odds ratio of T2D for each *α*‐diversity index of gut antibiotic resistome (in SD unit). Logistic regression models were adjusted for age, sex, BMI, smoking status, drinking status, education attainment, household income level, physical activity, Bristol stool scale, sequencing depth and microbial gene richness (MGR). The error bars represent confidence intervals. * *p* < 0.05.

In total, 639 bacterial species were identified in the 1210 samples. To focus on more representative species, 156 were kept for downstream analysis after filtering by relative abundance (mean> 0.01%) and prevalence (>10%) (Figure S2 and Table S4, Supporting Information). Pearson's correlation analyses indicated a positive association between *α*‐diversity of the gut antibiotic resistome and microbial gene richness (MGR) which is an *α*‐diversity indicator of the gut microbiota (*r* = 0.27–0.29, *p* < 2.2 × 10^−16^) (Figure S6A,B, Supporting Information). Similarly, Procrustes analysis demonstrated a strong cooperativity of the gut antibiotic resistome and gut microbiota profiles (Figure S6C, Supporting Information). Notably, our data showed that MGR was inversely associated with prevalent T2D after multivariable adjustment (Odds Ratio (OR) = 0.67, *p* < 0.001) (**Figure** **3**C). Therefore, we further performed a multivariable logistic regression analysis to investigate the association between *α*‐diversity of the gut antibiotic resistome and T2D, adjusted for MGR and other potential confounders. We found that larger Shannon index (OR = 1.19, 95% CI 1.01–1.41, *p* = 0.036) and observed richness (OR = 1.19, 95% CI 1.01–1.41, *p* = 0.038) of ARGs were associated with a higher risk of T2D (Figure 3C), which was also independent of microbial taxonomy (Table S5, Supporting Information), such as metformin‐related taxa *Proteobacteria* and *Escherichia*.^[^ ^9^ ^]^ Moreover, there were significant shifts for the gut antibiotic resistome composition across the healthy, prediabetes and T2D individuals (Bray‐Curtis distance, PERMANOVA, *p* < 0.05) (Figure 3A,B; Figure S7, Supporting Information). Interestingly, we observed a significant difference in the composition of gut ARG subtypes (*p* = 0.029) between healthy and prediabetes participants, but no significant change in the gut microbiota composition between the two groups (*p* = 0.304) (Table S6, Supporting Information).

### 2.3 Shifts in Gut Antibiotic Resistome and Microbial Species with Diabetes Status

**Figure 4:**
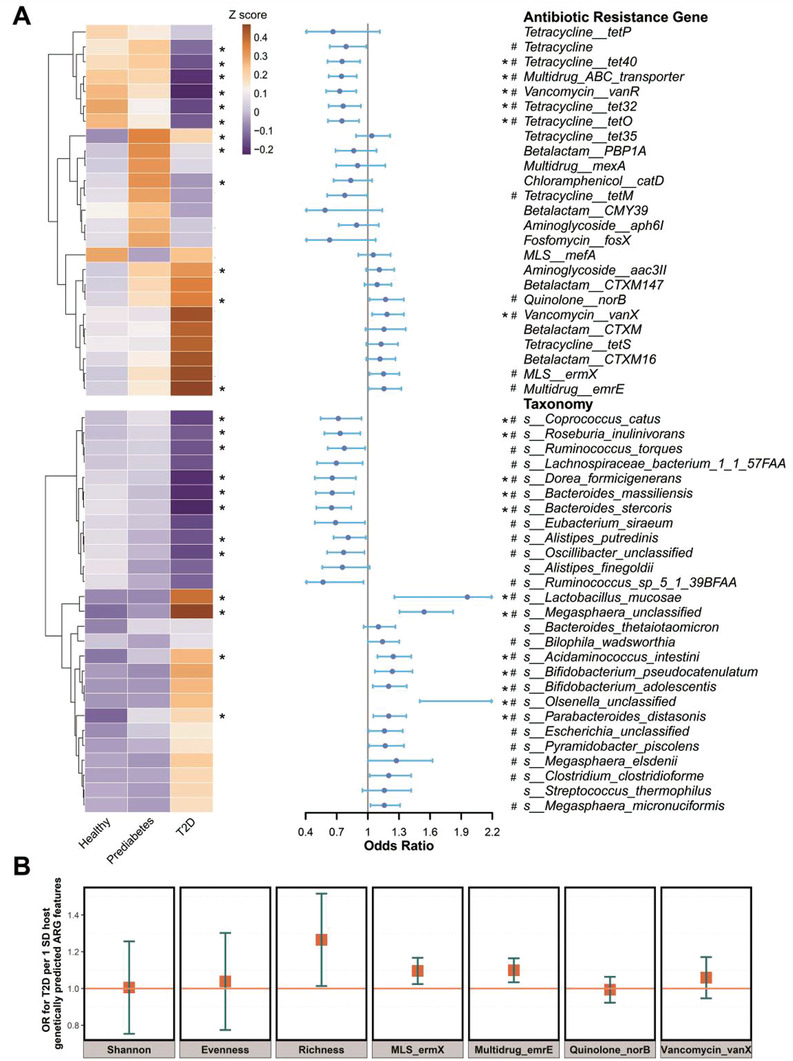
Shifts in gut antibiotic resistome and microbial species with diabetes status. A) Left heatmap shows the mean relative abundance (scaled by z score normalization) of the 25 identified T2D‐related ARGs and 27 identified T2D‐related microbial species from LASSO model. The abundance differences of these markers in healthy, prediabetes and T2D groups were examined by Kruskal–Wallis test. Right forest plot shows the odds ratio of T2D for each marker. Logistic regression models were performed, adjusted for age, sex, BMI, smoking status, drinking status, education attainment, income level and physical activity. The error bars represent confidence intervals. B) Mendelian randomization analysis (*n* = 947). Forest plot shows the odds ratios (95% confidence intervals) of T2D (outcome) per standard deviation increase in the host genetically predicted levels of the above identified ARG features (exposure). *p* values were corrected by Benjamini‐Hochberg method. MLS, Macrolide‐Lincosamide‐Streptogramin. *FDR‐corrected *p* < 0.05. #raw *p* < 0.05.

We then identified 25 ARGs and 27 microbial species related to T2D, based on the least absolute shrinkage and selection operator (LASSO) regression model (**Figure** **4**A; Tables S7 and S8, Supporting Information).^[^ ^10^ ^]^ Considering the progression effects of T2D, we combined the selected features from three binary dependent variable models: non‐T2D (healthy and prediabetes)/T2D, healthy/T2D, and prediabetes/T2D (Figures S8 and S9, Supporting Information). Among those markers, we observed a different overall pattern shift in the abundance for gut ARGs and microbial species from healthy, prediabetes to T2D. Specifically, the changes of abundance in 25 ARGs were divided into three distinct clusters while 27 microbial species were divided into two clusters (Table S9, Supporting Information). Among the ARGs, *Vancomycin_vanX*, *Multidrug_emrE*, *MLS_ermX* and *Quinolone_norB* were positively associated with T2D risk (OR = 1.15–1.18, *p* < 0.05) (Figure 4A; Table S10, Supporting Information). Moreover, the former two also showed a positive correlation with fasting blood glucose (FBG) (FDR‐corrected *p* < 0.05, Table S11, Supporting Information). We then used a one‐sample Mendelian randomization (MR) analyses to explore the potential causal association of the above identified ARG features with T2D. To enable the MR study, we performed genome‐wide association analyses for the selected ARG features and constructed genetic instruments for these features (Table S12, Supporting Information). We found that genetically predicted higher abundances of *Multidrug_emrE* and *MLS_ermX* were associated with higher T2D risk (OR = 1.10, 95% CI 1.04–1.16, *p* = 0.004; OR = 1.10, 95% CI 1.03–1.17, *p* = 0.01; Figure 4B). We also observed that genetically predicted higher levels of gut ARG richness were associated with higher T2D risk (OR = 1.26, 95% CI 1.00–1.50, *p* = 0.07, Figure 4B).

### 2.4 Gut Antibiotic Resistome Features are Associated with Cardiometabolic Risk Factors

**Figure 5:**
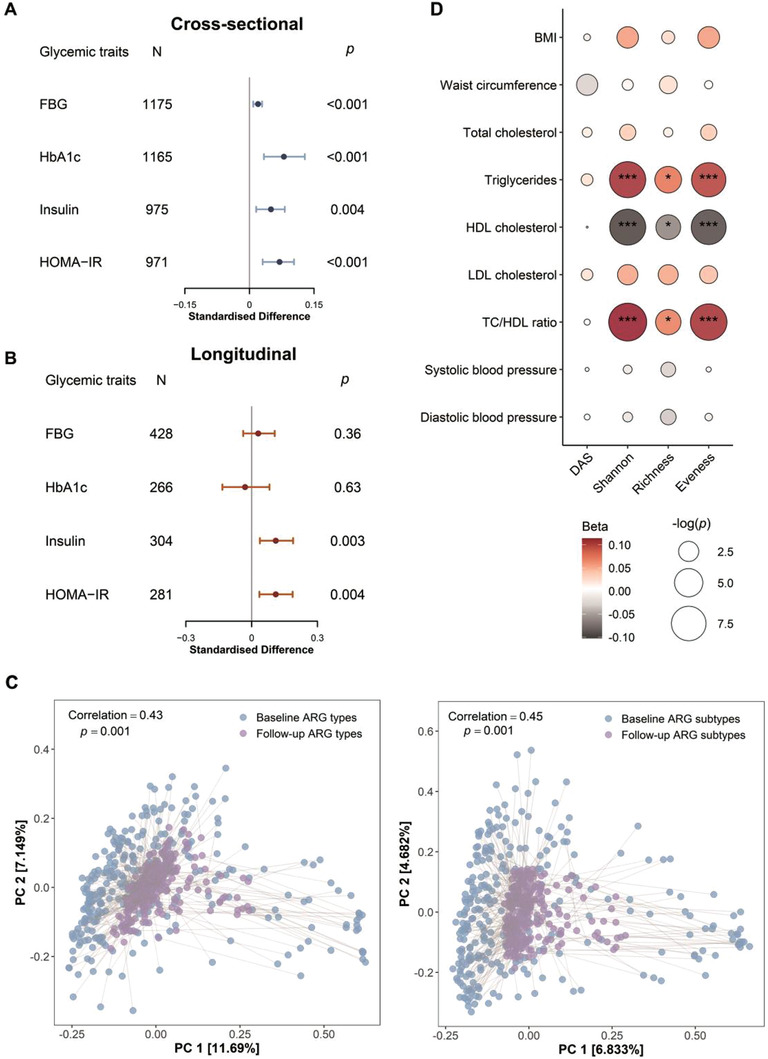
Gut antibiotic resistome features are associated with glycemic and cardiometabolic traits. A) Forest plot showing the cross‐sectional associations between DAS and glycemic traits. Linear regression models were performed, adjusted for age, sex, BMI, smoking status, drinking status, education attainment, income level, physical activity and Diabetes‐Microbiota Score (DMS). Standardized difference is the difference (in SD unit of the glycemic traits) per 1 SD change of DAS. The error bars represent confidence intervals. B) Forest plot showing the longitudinal associations between the baseline DAS and glycemic traits (repeated measured at baseline and a follow‐up visit). Linear mixed models were performed, adjusted for potential covariates the same as above linear model. Standardized difference is the difference (in SD unit of the glycemic traits) per 1 SD change of DAS. The error bars represent confidence intervals. C) Procrustes analysis of gut ARGs at the baseline versus gut ARGs at a follow‐up visit (*n* = 278). The median follow‐up time was 3.2 years. Baseline and follow‐up visit ARGs are shown as blue and purple dots, respectively. Baseline and follow‐up visit ARGs from the same individual are connected by grey lines. D) Associations between gut antibiotic resistome features and cardiometabolic risk factors. Linear regression models were used, adjusted for potential covariates the same as above linear model. Total number of participants in each analysis was 1175 for HDL cholesterol, LDL cholesterol, total cholesterol and TC/HDL ratio, 1176 for triglycerides, 1210 for diastolic blood pressure, systolic blood pressure and BMI, and 1203 for waist circumference. DAS, Diabetes‐ARG Score, FBG, insulin, HOMA‐IR were log‐transformed. FBG, fasting blood glucose, HbA1c, glycated hemoglobin; HOMA‐IR, homeostatic model assessment of insulin resistance; BMI, body mass index; HDL, high‐density lipoprotein; LDL, low‐density lipoprotein. HbA1c, glycated hemoglobin. Triglycerides and TC/HDL ratio were log‐transformed. *p < 0.05.

Based on the collection of T2D‐related gut ARGs, we constructed a novel Diabetes‐ARG Score (DAS) which was positively associated with T2D (*p* = 6.1 × 10^−5^), to represent the T2D‐related gut antibiotic resistome. In the sensitivity analysis, the granular control for T2D medication use or microbial taxonomies did not substantially change the association between DAS and T2D (Table S13, Supporting Information). We also found that the DAS was significantly associated with glycemic traits, such as FBG, glycated hemoglobin (HbA1c), insulin, and homeostatic model assessment of insulin resistance (HOMA‐IR). For instance, the multivariable linear regression model indicated that a higher DAS was associated with a higher level of FBG and HOMA‐IR (all *p* < 3.2 × 10^−4^) (**Figure** **5**A). Furthermore, we used a linear mixed model to investigate the longitudinal associations between DAS and glycemic traits after excluding the baseline T2D cases. Our analyses further validated the positive association of DAS with insulin and HOMA‐IR (both *p* < 5 × 10^−3^), suggesting that baseline gut antibiotic resistome was associated with T2D progression, characterized by the alteration of insulin resistance (Figure 5B).

The Procrustes analysis in 278 participants of the cohort showed that the gut antibiotic resistome had a significant consistency between the two time points (baseline and a follow‐up visit) (Figure 5C). Moreover, we also observed associations between gut antibiotic resistome features and other cardiometabolic traits. The gut ARG *α*‐diversity indices were positively associated with triglycerides and total cholesterol/high‐density lipoprotein (HDL) cholesterol ratio, while inversely associated with HDL cholesterol (Figure 5D; Tables S14 and S15, Supporting Information). Further subgroup analysis confirmed that the former two associations were consistent in both T2D or Non‐T2D participants (Table S16, Supporting Information), which indicated that these associations were independent of the T2D status. Overall, our findings revealed that a higher gut ARG *α*‐diversity was associated with a higher cardiometabolic risk.

### 2.5 Co‐Occurrence Patterns among T2D‐Related ARGs and Microbial Species

**Figure 6:**
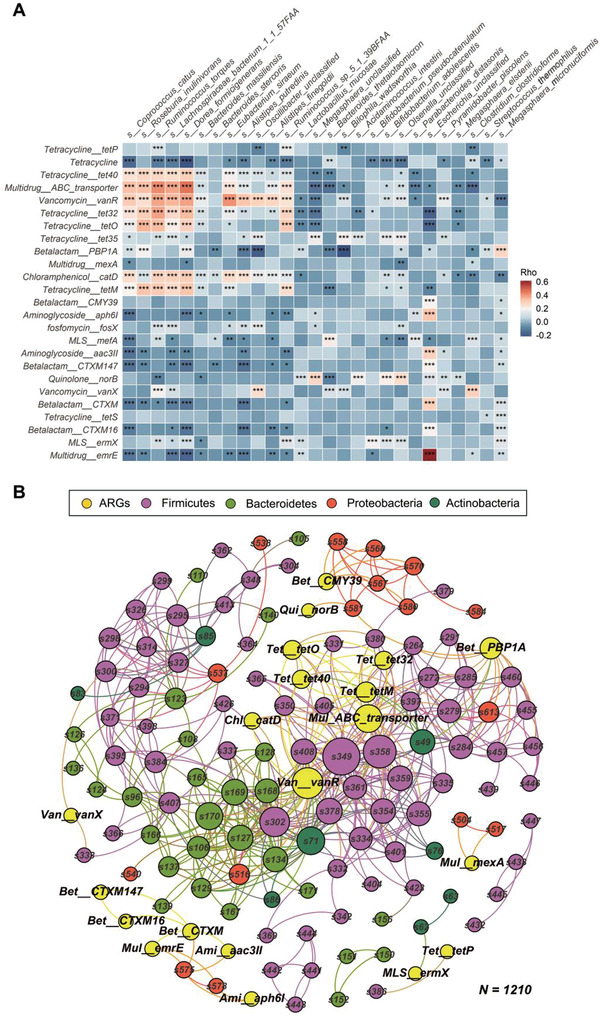
Co‐occurrence patterns among T2D‐related ARGs and microbial species. A) The heatmap shows the Spearman correlation coefficients between T2D‐related gut antibiotic resistance genes (ARGs) and T2D‐related microbial species. B) Correlation‐based networks of co‐occurring T2D‐related ARGs and all microbial species colored by node affiliation. A node stands for an ARG type/subtype or a species and a connection (i.e. edge) stands for a significant (FDR‐corrected *p* < 0.05, Spearman's rho ≥ 0.3, *n* = 1210) pairwise correlation. Node size is proportional to the number of connections (i.e., degree). Network was colored by ARGs and phylums. *Ami*, *Aminoglycoside*; Bet, *Betalactam*; *Chl*, *Chloramphenicol*; *MLS*, *Macrolide‐Lincosamide‐Streptogramin*; *Mul*, *Multidrug*; *Qui*, *Quinolone*; *Tet*, *Tetracycline*; *Van*, *Vancomycin*; *FDR‐corrected *p* < 0.05, ** FDR‐corrected *p* < 0.01, ***FDR‐corrected *p* < 0.001.

The positive correlations between the T2D‐related ARGs and microbial species suggest that the inverse correlations between ARGs and T2D may be caused by the depletion of beneficial bacteria. For instance, *Multidrug_ABC_transporter*, *Vancomycin_vanR*, and *Tetracycline*_*tet32* were positively associated with *Roseburia inulinivorans* which is a producer of butyric acid (Spearman's *rho* = 0.34, 0.25, and 0.3, FDR‐corrected *p* < 1.9 × 10^−17^) (**Figure** **6**A; Table S17, Supporting Information). A prior study showed that *R. inulinivorans* tended to be depleted in the gut microbiome of individuals with T2D, which was in line with our study (Figure 4A).^[^ ^5^ ^]^ Moreover, co‐occurrence network depicted the positive associations (Spearman r ≥ 0.3 and FDR‐corrected *p* < 0.05) between the T2D‐related ARGs and all of the bacteria species, which could be an effective way to track their potential hosts and co‐occurring bacteria of human gut or environmental ARGs.^[^ ^11^ ^]^ Specifically, both our data and the literature confirmed that *Escherichia* (s575 and s578) carries the *Multidrug_emrE* (Figure 6B; Tables S18–S21, Supporting Information).^[^ ^12^ ^]^ Here, T2D was first found to be positively associated with *Multidrug_emrE* (OR = 1.16, 95% CI 1.01–1.32, *p* = 0.037). Our data also showed that the number of edges of the ARG‐Species association networks had a gradual increment from heathy (87) to prediabetes (100) and T2D (128), suggesting that gut ARGs might be observed in more bacteria species with the disease progression (Table S22, Supporting Information). The above results together revealed a close relationship between the gut antibiotic resistome and T2D progression.

Plasmid‐mediated quinolone resistance in *Klebsiella pneumonia* was discovered in 1998, and it could transfer low‐level quinolone resistance to other bacteria.^[^ ^13^ ^]^ Here, the *Quinolone_norB* was only significantly co‐occurred with *Klebsiella pneumonia* (s581) in healthy and prediabetes populations (Figure S10, Supporting Information). However, in the T2D group this gene was also found to newly co‐occur with an unclassified *Olsenella* species (s89) which had a positive association with T2D (Figure 4A). In the global co‐occurrence networks that contain all the ARGs and microbial species, we found that ARGs tended to co‐occur more likely (O/R ratio = 1.67) with T2D‐positively related microbial species (T2D‐pos‐Spe) whereas less likely (O/R ratio = 0.21) with T2D unrelated species (ARG‐Non‐T2D‐Spe) than expected under random association (Table S23, Supporting Information). In addition, the co‐occurrence pattern between ARGs and T2D‐pos‐Spe tended to be randomized in prediabetes populations (as suggested by O/R ratio = 0.89), implying the potential for the spread of ARGs during the period of prediabetes, an early developmental stage of T2D.

### 2.6 Gut Antibiotic Resistome Features are Associated with Fecal Metabolome

Antibiotic resistance in bacteria is often associated with a metabolic burden, while microbial metabolic adaptations accompanying the development of antibiotic resistome in T2D patients are unclear.^[^ ^14^ ^]^ We therefore examined the associations between gut antibiotic resistome features and 117 targeted fecal metabolites. As expected, the gut antibiotic resistome was widely associated with the fecal metabolites. Specifically, we found that DAS and *Vancomycin_vanX* were positively associated with L‐isoleucine and L‐leucine (all FDR‐corrected *p* < 0.01) (Figure S11, Supporting Information). In addition, we observed that DAS and *Multidrug_emrE* were positively associated with 10‐trans‐heptadecenoic acid and 8,11,14‐eicosatrienoic acid, while negatively associated with butyric acid (Figure S11, Supporting Information). After the adjustment for 3 taxonomic principal components, more than a third of the significant associations were not substantially changed (Table S24, Supporting Information).

## 3 Discussion

Here, we not only demonstrate the previously unperceived association between ARGs and microbiota, but also discover a shift and spread in gut antibiotic resistome with the progression of T2D, which may accompany with widespread host‐microbial metabolic adaptations. Our study unravels a novel link between the gut microbiome and progression of T2D via antibiotic resistome.

A previous small‐scale study showed that *TetQ* and *ErmB* were the top two abundant ARGs in the Chinese.^[^ ^15^ ^]^ Similarly, we observed that *Tetracycline_TetQ* was also the most abundant ARG subtype in our cohort participants, and *MLS_ermB* was the third abundant ARG subtype. Moreover, six of the top ten abundant ARG subtypes were consistent with the study aforementioned.

Notably, we found for the first time, to the best of our knowledge, that ARG diversity was positively associated with T2D. In contrast, previous studies showed that the diversity of gut microbiota is inversely associated with T2D.^[^ ^16^ ^]^ To avoid potential confounding effect of the gut microbiota, our model was adjusted for the total microbial gene richness and taxonomies. These results suggest that the diversity of the gut antibiotic resistome is different from that of the gut microbiota, in terms of their relationships with metabolic disease. In addition, we observed a significant difference in the composition of gut ARG subtypes between healthy and prediabetes participants, but no significant change in the gut microbiota composition between the two groups. The latter is in line with a previous study which reported no significant microbiota change between the impaired fasting glucose tolerance participants and the low‐risk normal glucose tolerance participants.^[^ ^17^ ^]^ These results suggest that the gut antibiotic resistome may change earlier than the gut microbiota during the progression of T2D, and/or that changes in the gut antibiotic resistome are more sensitive to the development of T2D.

Although it is hard to determine whether the gut ARG features causally increase the T2D risk, our data indirectly permit fairly speculation. For example, the results of MR analyses in our study reveal a potential causal links between gut ARG richness, *Multidrug_emrE* and T2D. *Multidrug_emrE* is a small‐drug efflux pump, which confers resistance to a wide variety of antimicrobial agents including cationic disinfects (e.g., quaternary ammonium compounds used in the hospitals and food industry)^[^ ^18^ ^]^ and antibiotics (e.g., ampicillin, erythromycin, and tetracycline).^[^ ^19^ ^]^ Nevertheless, the current MR analyses were preliminary and exploratory, given the limited availability of genetic instrument for analyzing ARG features. More studies are required to further elucidate the causal links between gut ARGs and T2D.

Previous evidence showed that plasma L‐isoleucine and L‐leucine had highly significant associations with future diabetes risk.^[^ ^20^ ^]^ In our study, both of them in fecal samples were positively associated with DAS and *Vancomycin_vanX*. Moreover, we observed that DAS and *Multidrug_emrE* were positively associated with 8,11,14‐eicosatrienoic acid, which is also known as dihomo‐gamma‐linolenic acid (DGLA). A cross‐sectional study revealed that the DGLA level was an independent determinant for HOMA‐IR in 225 Japanese patients with T2D.^[^ ^21^ ^]^ Furthermore, our study found that the gut antibiotic resistome was widely associated with the fecal metabolites, which may reflect the host‐microbial metabolic adaptation. Bacteria could develop resistance to many classes of antibiotics vertically, by making mutations in central housekeeping genes, which correspondingly affected metabolism.^[^ ^22^ ^]^ One study on *E. coli* demonstrated that the acquisition of antibiotic resistance is accompanied by specifically reorganized metabolic networks in order to circumvent metabolic costs.^[^ ^23^ ^]^ Taken together, these results provide a potential interpretation for the mechanism behind the observed association in the present study.

The present study has several strengths. First, we profile the gut antibiotic resistome configuration in a large population with different diabetes status, which has not been investigated before. Second, previous studies mainly focus on the relationship between gut microbiota and T2D, while we also examine the association between gut antibiotic resistome and T2D progression, and the results were validated by the longitudinal analyses. Finally, we modelled, for the first time, the association between the antibiotic resistance and T2D and constructed a novel diabetes‐ARG score, which may help inform a new mechanism discovery for the pathophysiology of T2D. Nonetheless, there are several limitations. First, our study is based on the Chinese population and may not be generalizable to other ethnicities. Therefore, the generalization of our findings would require validation in other countries or ethnicities. Second, our results are from an observational study, which is subject to the influence of residual confounders. Third, given the considerable effects of drugs on gut microbiome,^[^ ^24^ ^]^ and the factor that diabetes patients are prone to infection, although we have controlled the taxonomic confounders for our findings, our results may still be affected by specific drugs exposures, such as antibiotics or metformin. Finally, detailed mechanism behind our observed association is not clear, more mechanistic investigation in future is needed to provide causality.

In conclusion, our study depicts a comprehensive profile of the gut antibiotic resistome in a large human cohort and provides a novel insight about the relationship between antibiotic resistance and T2D progression. These results also suggest that the gut antibiotic resistome is closely connected with fecal metabolites and host metabolic health. These novel ARG features may potentially serve as intervention target of T2D in future studies.

## 4 Experimental Section

### Study Design and Participants

The current study was based on the Guangzhou Nutrition and Health Study (GNHS) which is an ongoing community‐based cohort study in China. Between 2008 and 2013, a total of 4048 participants aged between 45 and 70 years, who lived in Guangzhou city for at least 5 years were enrolled in the GNHS. There were two waves for recruitment, 3169 were recruited between 2008 and 2010, and 879 were recruited between 2012 and 2013. All participants were followed up approximately every 3 years. Detailed information on the study design has been reported previously.^[^ ^25^ ^]^ The study was registered at clinicaltrials.gov (NCT03179657).

In the present study, participants were excluded according to the criteria: without metagenomics data (*n* = 2829), with missing data on main covariates including age, sex, BMI, smoking status, alcohol drinking, education, and income level (*n* = 2) or without Bristol stool scale information (*n* = 1). Participants who used antibiotics within two weeks before fecal samples collection were also excluded (*n* = 6). Finally, 1210 participants were included in the analysis (Figure S1, Supporting Information). The study protocol was approved by the Ethics Committee of the School of Public Health at Sun Yat‐sen University (2018048) and Ethics Committee of Westlake University (20190114ZJS0003). All participants provided written informed consent.

### Metadata Collection—Assessment of Diabetes Status and Definitions

Diabetes status was defined according to the criteria of the American Diabetes Association and the World Health Organization: prediabetes was ascertained if a participant met one of the following criteria: i) without a history of diabetes,(ii) glycated hemoglobin (HbA1c): 5.7–6.4%, iii) fasting blood glucose (FBG): 6.1–6.9 mmol L^−1^; T2D cases were defined in adults as meeting one of the criteria: FBG ≥ 7.0 mmol L^−1^, HbA1c ≥ 6.5% or self‐reported medical treatment for diabetes.^[^ ^26^ ^]^ The diabetes medical history was from the questionnaire: “Have you taken T2D medications following your physicians’ instructions during the past year?

1: Yes; 2: Most of time (Yes); 3: Half of the time (Yes); 4: Little of the time (Yes); 5: No”. For those having record of taking diabetic medications, they were defined as diabetes cases in the study.

### Metadata Collection—Covariates Assessment

The metadata on demographics, lifestyle, medical history and physical activity was collected by questionnaires. Education attainment was categorized into primary (0–6 years), secondary (7–9 years), and higher education (≥ 10 years). Smoking status was categorized into current smoker and non‐smoker. Alcohol drinking was classified as current drinker and non‐drinker. Habitual dietary intakes at baseline were estimated from a validated food frequency questionnaire (FFQ), which recorded the frequencies of foods in the past 12 months. The food intakes were then divided into 16 food groups according to the Guidelines for Measuring Household and Individual Dietary Diversity.^[^ ^27^ ^]^

Physical activity was assessed as total metabolic equivalent for task (MET) hours per day based on a validated physical activity questionnaire, and it was classified into four groups according to quartiles. Body weight, height, waist circumference, hip circumference, neck circumference, and blood pressure were measured by trained nurses on site. Fasting venous blood samples were collected at both baseline and follow‐up visits. Glucose, total triglycerides, high‐density lipoprotein (HDL) cholesterol, low‐density lipoprotein (LDL) cholesterol, and total cholesterol in serum were measured on an automated analyzer (Roche cobas 8000 c702, Shanghai, China). Glycated hemoglobin (HbA1c) was measured with the Bole D‐10 Hemoglobin A1c Program on a Bole D‐10 Hemoglobin Testing System. Insulin was measured using the electrochemiluminescence immunoassay (ECLIA, Roche cobas 8000 e602) method. The homeostatic model assessment of insulin resistance (HOMA‐IR) was calculated as fasting blood glucose (mmol L^−1^) times fasting insulin (mIU L^−1^) divided by 22.5.^[^ ^28^ ^]^

### Fecal Metagenomics Profiling

Fecal samples of 1210 participants were collected during on‐site study visits between 2015 and 2019. Before DNA extraction, the fecal samples were kept frozen at −80 °C. Fecal DNA extractions were carried out by a standardized CTAB procedure. DNA concentration was measured using Qubit dsDNA Assay Kit in Qubit 2.0 Fluorometer (Life Technologies, CA, USA). For DNA library preparation, a total amount of 1µg DNA per sample was used as input material. In addition, the NEBNext Ultra DNA Library Prep Kit (NEB, USA) was used following manufacturer's recommendations and index codes were added to attribute sequences to each sample. The DNA samples were fragmented by sonication to a size of ≈350 bp. Then, the DNA fragments were end‐polished, A‐tailed, and ligated with the full‐length adaptor for Illumina sequencing with further PCR amplification. After that, PCR products were purified (AMPure XP system) and libraries were analyzed for size distribution by Agilent2100 Bioanalyzer and quantified using real‐time PCR. The clustering of the index‐coded samples was performed on a cBot Cluster Generation System according to the manufacturer's instructions. After cluster generation, the library preparations were sequenced on an Illumina HiSeq platform and 150 bp paired‐end reads were generated. Finally, on average, 42.4 million paired‐end raw reads for each sample were obtained(Table S2, Supporting Information).

Next, raw sequencing reads were first quality‐controlled with PRINSEQ (v0.20.4): 1) trim the reads by quality score from the 5′ end and 3′ end with a quality threshold of 20; 2) remove read pairs when either read was < 60 bp, contained “N” bases or quality score mean bellow 30; and 3) deduplicate the reads. Reads that could be aligned to the human genome (H. sapiens, UCSC hg19) were removed (aligned with Bowtie2 v2.2.5 using –reorder –no‐contain –dovetail).^[^ ^29^ ^]^

Taxonomic profiling of the shotgun metagenomic data was performed using MetaPhlAn2 (v2.6.02), which uses a library of clade‐specific markers to provide pan‐microbial quantification at the species level.^[^ ^30^ ^]^ MetaPhlAn2 was run using default settings. Only species‐level relative abundance data were considered in this study. Species were filtered out if their mean relative abundance and prevalence were <0.01% or <10% (Figure S2, Supporting Information). Functional profiling was performed with HUMAnN2 v2.8.1, which maps sample reads against the sample‐specific reference database to quantify gene presence and abundance in a species‐stratified manner, with unmapped reads further used in a translated search against Uniref90 to include taxonomically unclassified but functionally distinct gene family abundances.^[^ ^31^ ^]^ Microbial gene richness (MGR) was computed by the number of genes present in each sample.

The ARG types and subtypes were annotated by ARGs‐OAP v2.0 with default parameters.^[^ ^32^ ^]^ ARG‐OAP is a read‐based tool, which was developed for the rapid annotation and classification of ARGs using SARG database and has been widely applied for antibiotic resistome profiling in human gut and environmental microbiota.^[^ ^33^ ^]^ Compared with the metagenome‐assembly‐based method, the read‐based approaches have a good scalability in the case of ever‐increasing query sequences and antimicrobial resistance reference data. More importantly, they were able to identify ARGs from low‐abundance organisms present in complex communities, which may be missed by assembly‐based methods owing to incomplete or poor assemblies.^[^ ^34^ ^]^ The expanded SARG database of ARGs‐OAP v2.0 contains sequences not only from CARD and ARDB databases, but also carefully selected and curated sequences from the latest protein collection of the NCBI‐NR database, to keep up to date with the increasing number of ARG deposited sequences. Each reference sequence was tagged with its functional gene annotation (ARG subtype) and membership within a class of antibiotics targeted by the gene (ARG type). For example, the prefix of *Tetracycline_TetQ* is ARG type and the suffix ‘*TetQ*’ is ARG subtype. The pipeline provides an algorithm for estimating cell number. The abundance of ARG was calculated and normalized by the cell number in the research, expressed as ‘copies of ARG per prokaryote's cell’ using following equation

(1) $$\text{ARG}\;\text{abundance}=\Sigma_{1}^{n}\frac{N_{\mathrm{ARG}}\times L_{\mathrm{reads}}/L_{\mathrm{ARG}-\mathrm{ref}}}{\text{cell}\;\text{number}}$$

where *n* is the number of ARG reference sequences belonging to an ARG type or subtype; *N*_ARG_ is the number of the ARG‐like sequences annotated to one specific ARG reference sequence in the metagenome; *L*_reads_ is the sequencing length of metagenomic reads; *L*_ARG‐ref_ is the average length of the correspondingly specific ARG reference sequences. The cell number was computed based on the following equation^[^ ^35^ ^]^

(2) $$\text{Cell}\;\text{number}=\frac{\Sigma_{1}^{n}N_{16S\;\text{sequences}}\times L_{\mathrm{reads}}/L_{16S\;\text{Sequences}}}{\Sigma_{i\;=\;1}^{m}M_{i}\times a_{i}/A}$$

where *m* is the total taxa detected from the metagenomics dataset based on the extracted hyper variable region information; *M_i_* represents the number of copies of taxon *i* from the CopyRighter database; *a_i_* is the number of aligned hypervariable sequences of taxon *i* in the metagenomics datas set; *A* is the total number of aligned hypervariable sequences in all *m* taxa.

In addition, the ARG types or subtypes were excluded that were present in less than 10% of the samples (Figure S2, Supporting Information). Unclassified ARG types or subtypes were also excluded in current study. The *α*‐diversity of gut antibiotic resistome was represented by three diversity indices: Shannon, Richness (Observed unique ARGs) and Evenness (Pielou's index), which was estimated by the *vegan* R package.^[^ ^36^ ^]^ To validate whether the annotation results were robust to differences in the sequencing depths across samples, top 50 and bottom 50 samples based on the reads number after quality control were selected. The reads number of these 100 samples were range from 18M to 61M. Then, every sample was randomly rarefied to 18M reads. The annotation pipeline was re‐ran to obtain the abundance and composition of ARGs. The alpha diversity measures of ARGs without rarefaction and with rarefaction were highly consistent (Pearson's r >0.96; Figure S3, Supporting Information).

### Genotyping Data

Host DNA was extracted from leukocytes using the TIANamp Blood DNA Kit (DP348, TianGen Biotech Co, Ltd., China) according to the manufacturer's instructions. DNA concentrations were determined using the Qubit quantification system (Thermo Scientific, Wilmington, DE, USA). Extracted DNA was stored at −80 °C. Genotyping was carried out with Illumina ASA‐750K arrays. Quality control and relatedness filters were performed by PLINK1.9.^[^ ^37^ ^]^ Individuals with a high or low proportion of heterozygous genotypes (outliers defined as 3 standard deviations) were excluded^[^ ^38^ ^]^. Individuals who had different ancestries (the first two principal components ± 5 standard deviations from the mean) or related individuals (IBD > 0.185) were excluded.^[^ ^38^ ^]^ Variants were mapped to the 1000 Genomes Phase 3 v5 by SHAP EIT,^[^ ^39^ ^]^ and then genome‐wide genotype imputation was conducted with the 1000 Genomes Phase 3 v5 reference panel by Minimac3.^[^ ^40^ ^]^ Genetic variants with imputation accuracy RSQR > 0.3 and MAF > 0.05 were included in the analysis.

### Targeted Fecal Metabolome Profiling

The absolute quantification of fecal samples (*n* = 1012) was performed by an ultra‐performance liquid chromatography coupled to tandem mass spectrometry (UPLC‐MS/MS) system. Detailed information about the measurements has been described previously.^[^ ^41^ ^]^ The list of metabolites was selected to capture the microbiota‐related metabolites and some key host metabolites. Finally, 117 metabolites were selected. These metabolites mainly include amino acids, bile acids and fatty acids.

### Statistical Analysis

All statistical analyses were performed using Stata version 15 or R version 4.0.2. Participants were categorized into three groups (healthy, prediabetes and T2D) based on their diabetes status. To explore the compositional variation of gut antibiotic resistome, 37 factors (including demography, physiology and dietary factors) were correlated to the ARG subtype distance matrix (Bray‐Curtis) using permutational multivariate analysis of variance (PERMANOVA). Then, Pearson correlation analysis was used to examine the association between *α*‐diversity of gut antibiotic resistome and microbial gene richness. The principal coordinates analysis (PCoA) on Bray‐Curtis distance and PERMANOVA were performed to examine the structural differences of gut antibiotic resistome and gut microbiota among three different groups using the *adonis* function (permutations = 999). In addition, Procrustes analysis was performed to investigate the relationship between gut antibiotic resistome and gut microbiota, and the *p* value was generated based on 999 permutations. Then the association between *α*‐diversity indices of gut antibiotic resistome and gut microbiota and prevalent T2D was examined using a logistic regression model, adjusted for potential confounders as follows: age, sex, body mass index (BMI), physical activity, smoking status, drinking status, education attainment, household income level, Bristol stool scale, sequencing depth, and MGR. The *α*‐diversity indices were z‐score normalized before regression analysis. As metformin showed impact on the abundance of human gut microbiome,^[^ ^9^ ^]^ to control the confounding effect of microbial taxonomies, sensitivity analysis was conducted for the PERMANOVA model and logistic regression model with or without adjustment for the taxonomies, such as the abundance of *Proteobacteria* (phylum), *Firmicutes* (phylum), *Escherichia* (genus) or three top taxonomic principal components (PCs). The three top PCs were obtained through the principal component analysis based on the whole species abundance matrix. These models were compared regarding the *p* values to examine the robustness of corresponding results.

To identify the markers of T2D, the least absolute shrinkage and selection operator (LASSO) regression model was used with 5 repeated 5‐fold cross‐validations based on the gut ARGs, microbial species and main covariates (age, sex, BMI, physical activity, smoking status, drinking status, education attainment, household income level, systolic blood pressure, diastolic blood pressure and Bristol stool scale). LASSO was implemented in the R package *glmnet* using a binomial response type for binary dependent variables (Non‐T2D (healthy and prediabetes)/T2D, healthy/T2D, prediabetes/T2D).^[^ ^42^ ^]^ The predictive performance of the selected models was assessed by estimating the area under the receiver operation curve (AUC) for binary responses (alpha = 1; 100 lambda tested) (Figure S8, Supporting Information). The selected value of ‘lambda.min’ was defined using cross‐validation, the lambda controls the overall impact of LASSO. Then the features were merged with nonzero coefficients of the three models (Non‐T2D/T2D, healthy/T2D, prediabetes/T2D) as markers of T2D progression.

Subsequently, the abundances of the markers were z score transformed. Kruskal‐Wallis test and Mann‐Whitney U test were used to examine the abundance differences of the marker ARGs and microbial species among healthy, prediabetes and T2D groups. The logistic regression was performed to estimate the odds ratio of T2D for the markers after adjustment for age, sex, BMI, smoking status, drinking status, education attainment, income level, and physical activity. We then performed a linear regression analysis to explore the associations between the T2D‐related ARGs and glycemic traits, such as FBG, HbA1c, insulin and HOMA‐IR, adjusted for the covariates as the above model. Here, *p* values were controlled by Benjamini‐Hochberg method for multiple tests. FDR‐corrected or raw *p* values < 0.05 were considered to be significant.

### Statistical Analysis—Genome‐Wide Association Analysis of T2D‐Related ARG Features

To further examine the probability that ARG features increased the risk of T2D, GWAS for ARG *α*‐diversity indices and T2D positively related ARG markers were conducted in 947 participants with both host genetic and metagenomics data. For the targeted ARG features, we used log transformation and z‐score normalization to change the skewed distribution before GWAS analysis. A mixed linear model‐based leave‐one‐chromosome‐out association (MLMA‐LOCO) analysis in GCTA was used to assess the association, fitting the first five genetic principal components of ancestry, age and sex as fixed effects and the effects of all the SNPs as random effects.^[^ ^43^ ^]^

### Statistical Analysis—One Sample MENDELIAN Randomization Analysis

To test if ARG features were causally linked to T2D, the genetic variants used for one sample MR analysis were extracted from the GNHS study with a moderate cutoff of *p* < 5 × 10^−5^. The weighted polygenic risk score for each trait was constructed with the effect size from the additive model. The two‐stage one‐sample analysis was implemented to estimate the potential casual association. The first stage included a regression of the ARGs or *α*‐diversity index on the polygenic risk score, adjusted for age at the time of stool sample collection, sex and the first five genetic principal components of ancestry. The second stage included a logistic regression of T2D using the prediction value constructed with the first stage regression, adjusted for age, sex and the first five genetic principal components of ancestry. Results were presented as odds ratio per 1‐SD increase in polygenic risk score.

Based on the identified T2D‐related ARGs, a diabetes‐ARG score (DAS) was constructed as a new feature to represent the gut antibiotic resistome associated with T2D. The formula was used to compute DAS as follows

(3) $$\mathrm{DAS}=\Sigma_{1}^{n}\left(OR_{i}-1\right)\;\times A_{i}$$

Where *n* is the number of marker ARGs of T2D progression; OR_i_ is the odds ratio of T2D for the i‐th marker ; A_i_ is the i‐th normalized abundance (z score) of ARGs.

To test the reliability of DAS, a logistic regression analysis was performed to examine the cross‐sectional association (independent variable and dependent variable were from the same time point) between DAS and T2D, adjusted for potential confounders. To clarify the influence of T2D medication use on the above model, the abundance of *Proteobacteria* (phylum), *Firmicutes* (phylum), and *Escherichia* (genus) was further separately or simultaneously adjusted. A sensitivity analysis was also performed after excluding the participants who used diabetic medications in the recent one year before stool sampling. The significances of DAS in these models were tested by the Wald test. In addition, the cross‐sectional correlation between DAS and glycemic traits, including fasting blood glucose, HbA1c, insulin and HOMA‐IR (homeostatic model assessment of insulin resistance), was assessed. The linear regression analysis was performed after adjustment for age, sex, BMI, smoking status, drinking status, education attainment, income level, physical activity. Considering that T2D related taxonomies of the gut microbiota may confound the above association, the Diabetes‐Microbiota Score (DMS) constructed by the same method as DAS was also adjusted, in the logistic regression model. Based on the identified T2D‐related species, DMS was constructed as a feature to represent the gut microbiota associated with T2D. The formula was used to calculate DMS as follows

(4) $$\mathrm{DMS}=\Sigma_{1}^{n}\left(OR_{i}-1\right)\;\times A_{i}$$

where *n* is the number of marker species of T2D progression; *OR_i_* is the odds ratio of T2D for the i‐th marker ; *A_i_* is the i‐th relative abundance of species.

Moreover, the linear mixed models were used to examine the longitudinal association between DAS (baseline) and glycemic traits (repeat measure at baseline and follow‐up visit) after excluding the baseline T2D cases, adjusted for age, sex, BMI, smoking status, drinking status, education attainment, income level, physical activity and DMS. Here, the independent variable was baseline DAS (single time point) and the dependent variables were glycemic traits (at two time points). As only the resistome information was used at baseline of the cohort, it might be that the gut antibiotic resistome would change over time. To address this concern, a Procrustes analysis was performed in 278 participants of the cohort with a median follow‐up of 3.2 years. The fecal samples of these participants were collected twice (baseline and a follow‐up visit). In addition, a multivariable linear regression model was used to assess the cross‐sectional association of the gut antibiotic resistome features (including DAS and *α*‐diversity indices) with other cardiometabolic risk factors, including BMI, waist circumference, total cholesterol, triglycerides, HDL cholesterol, LDL cholesterol, TC/HDL ratio, systolic blood pressure, and diastolic blood pressure. The dependent variables with skewed distribution were log‐transformed before analysis (fasting blood glucose, insulin, HOMA‐IR, TC/HDL‐C and TG). A subgroup analysis was also performed in T2D (cases) or Non‐T2D participants (controls). The regression associations were expressed as the difference in cardiometabolic risk factors (in SD unit) per 1 SD difference in each gut antibiotic resistome feature. The significance was assessed by the two‐tailed *t* test. *P* values were corrected for multiple testing using the Benjamini‐Hochberg procedure.

### Statistical Analysis—Network Analysis of ARG‐Microbe Associations

Spearman correlation analysis was performed to examine the associations between T2D‐related ARGs and T2D‐related gut microbial species, based on ‘Co‐occurrence Network Analysis’ package (github.com/RichieJu520)^[^ ^44^ ^]^. To explore the underlying associations among T2D‐related ARGs and all gut microbial species, a correlation matrix was constructed by calculating the pairwise Spearman correlation coefficients. A correlation between ARG‐ARG, species‐species, or ARG‐species was considered significant if FDR‐corrected *p* < 0.05. Gephi was further applied to visualize the correlations (Spearman's rho was ≥ 0.3) in a network interface and explore its topological properties.

To fully explore the hidden deterministic (or non‐random) co‐occurrence patterns, the global co‐occurrence associations between all the gut ARGs and microbial species identified were also computed. The observed (O%) and random incidences (R%) of co‐occurrence correlation between two group entities (i.e., ARG and/or species) were statistically checked using the method as described previously.^[^ ^44^, ^45^ ^]^ Briefly, O% was calculated as the number of observed edges divided by total number of edges in the observed network, while R% was theoretically calculated by considering the frequencies of two group entities and assuming random association. Here co‐occurrence patterns with Spearman's rho ≥0.6, O% ≥1.0/R% ≥1.0, and O/R ≥1.5/O/R ≤ 0.5 were considered as significant difference.

Spearman correlation analysis was finally used to investigate the associations between gut antibiotic resistome features (DAS, *Multidrug_emrE*, *Vancomycin_vanX*, *Quinolone_norB*, and *MLS_ermX*) and 117 fecal metabolites. The concentrations of the metabolites were transformed to z‐scores before analysis. To evaluate whether these associations are independent of the taxonomic shifts, a linear regression model was used to examine the association of 3 taxonomic PCs with log‐transformed concentrations of metabolites. Residuals of the model were taken as the taxonomy‐adjusted metabolites for Spearman correlation analysis with gut antibiotic resistome features. The Spearman's rank correlation test was used to assess their significance. *P* values were corrected for multiple testing using the Benjamini‐Hochberg procedure.

## Conflict of Interest

The authors declare no conflict of interest.

## Author Contributions

M.S., G.Z., F.‐f.Z., and Y.F. contributed equally to this work. J.S.Z., F.J., Y.M.C. designed research. C.W.L., F.X. collected the data. M.L.S., G.Q.Z., F.J., and L.Y. performed the data analysis. M.L.S., F.J., and J.S.Z. wrote the manuscript (M.L.S. drafted the initial manuscript. J.S.Z., F.J., G.Q.Z., and M.L.S. finalized the manuscript). J.S.Z., Y.M.C., F.J., Y.Q.F., G.Q.Z., X.X.L., Z.M., and Z.J. revised the manuscript. J.S.Z. and F.J. had primary responsibility for the final content; All authors read, revised, and approved the final manuscript.

## Code Availability Statement

The scripts and instructions used for metagenomics processing pipeline are available from https://github.com/emblab‐westlake/rMAP. For ARGs annotation, we used the open‐source ARG‐OAP2 pipeline, which is available at https://github.com/biofuture/Ublastx_stageone.

## Patient and Public Involvement

Patients and/or the public were not involved in the design, or conduct, or reporting, or dissemination plans of this research.

## Supporting information

Supporting Information

Supplemental Table 1

## Data Availability

The data that support the findings of this study are openly available in CNGBdb at https://db.cngb.org/search/project/CNP0001510/, reference number 1510.
