# Supplementary material for: Human Gut Antibiotic Resistome and Progression of Diabetes
Source: Adv Sci (Weinh). 2022 Feb 10;9(11):2104965. doi: 10.1002/advs.202104965 (PMC9008416; doi:10.1002/advs.202104965)

ADVANCED SCIENCE
Open Access

Supporting Information

for *Adv. Sci.*, DOI: 10.1002/advs.202104965

Human gut antibiotic resistome and progression of diabetes

Menglei Shuai, Guoqing Zhang, Fang-fang Zeng, Yuanqing Fu, Xinxiu Liang, Ling Yuan, Fengzhe Xu, Wanglong Gou, Zelei Miao, Zengliang Jiang, Jia-ting Wang, Lai-bao Zhuo, Yu-ming Chen, Feng Ju*, Ju-Sheng Zheng**

Supplementary Materials

Human gut antibiotic resistome and progression of diabetes

Menglei Shuai[1,2#], Guoqing Zhang[3,4#], Fang-fang Zeng[5,6#], Yuanqing Fu[1,2,7#], Xinxiu Liang[1,2], Ling Yuan[3,4], Fengzhe Xu[1,2], Wanglong Gou[1,2], Zelei Miao[1,2], Zengliang Jiang[1,2,7], Jia-ting Wang[5], Lai-bao Zhuo[5], Yu-ming Chen[5*], Feng Ju[2,3,4*], Ju-Sheng Zheng[1,2,7*]

This word file includes:

Figs. S1 to S11

Fig. S1. Flow diagram of participants' selection for the analyses of present study.

Fig. S2. Workflow for analysis of metagenomics sequencing data.

Fig. S3. Pearson's correlation of the alpha diversity between non-rarefaction and rarefaction (n = 100).

Fig. S4. Abundance and prevalence of gut antibiotic resistome in GNHS.

Fig. S5. The effect sizes of host factors in gut antibiotic resistome grouped by diabetes status.

Fig. S6. Correlations between gut antibiotic resistome diversity and microbiota.

Fig. S7. The comparison of inter-individual Bray-Curtis distance of gut antibiotic resistome.

Fig. S8. The performance of models based on LASSO feature selection.

Fig. S9. Veen plot of biomarkers identified by LASSO models.

Fig. S10. Networks of co-occurring T2D-related ARGs and gut microbiota, grouped by diabetes status.

Fig. S11. Associations between gut antibiotic resistome features and fecal metabolites (n = 1012).

Fig. S1. Flow diagram of participants' selection for the analyses of present study.

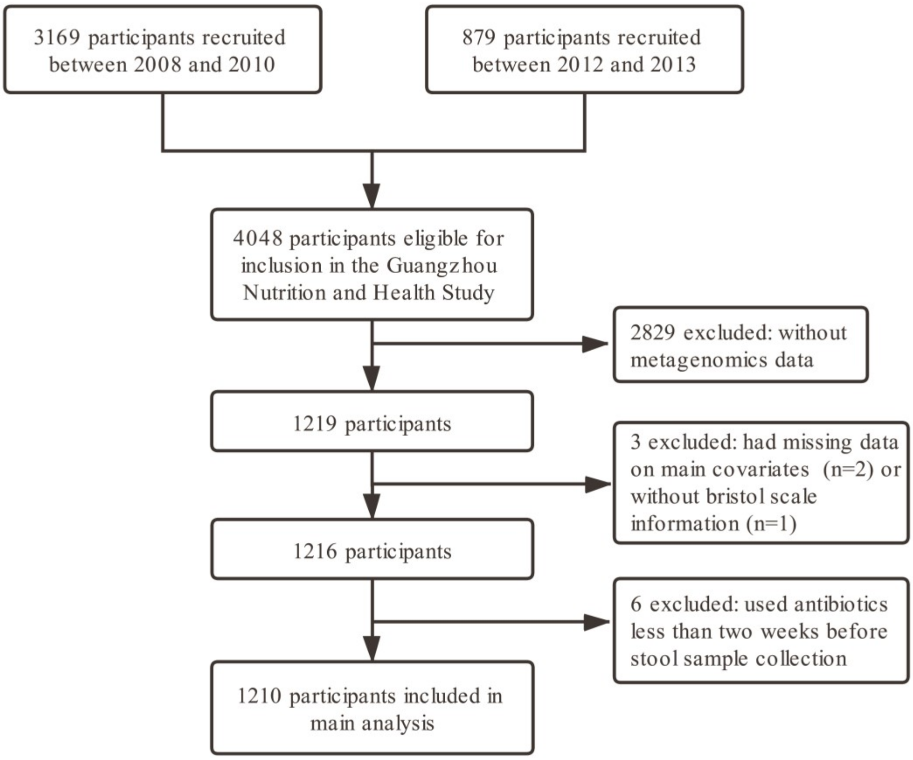

Fig. S2. Workflow for analysis of metagenomics sequencing data. Two distinct but complementary pipelines were used for metagenomics analysis. 19 antibiotic resistance gene (ARG) types and 805 ARG subtypes were annotated using ARG-OAP2. 639 microbial bacteria species were identified using MetaPhlAn2. PCoA, principal coordinates analysis.

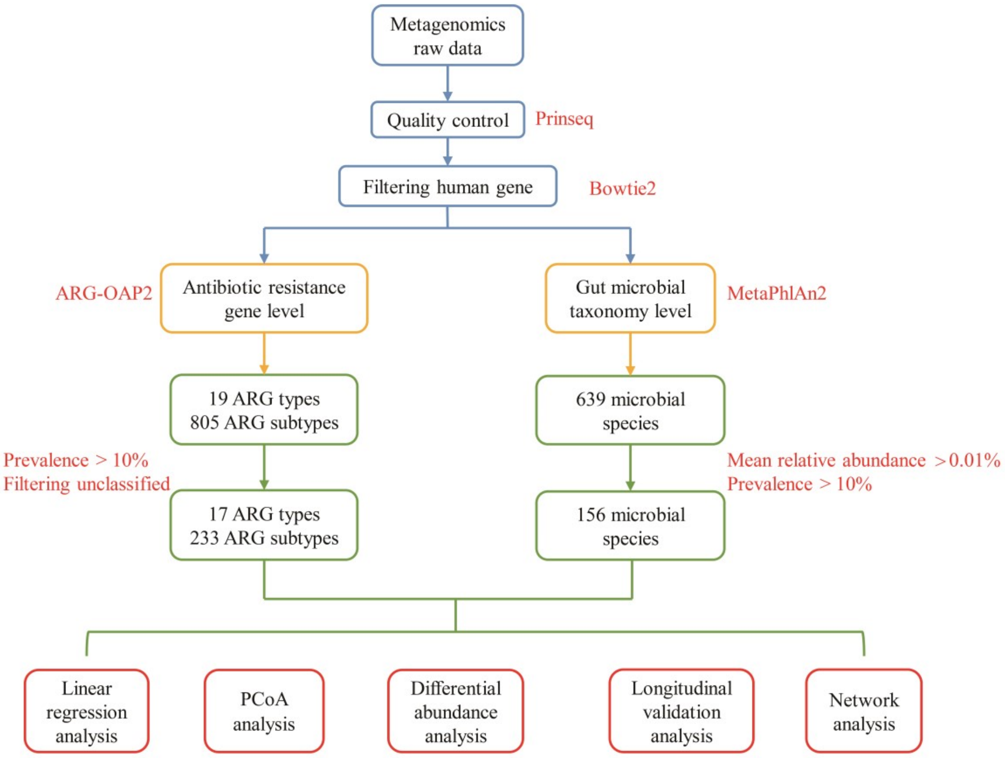

Fig. S3. Pearson's correlation of the alpha diversity between non-rarefaction and rarefaction (n = 100). The dot plots show the Pearson's correlation of the alpha diversity between non-rarefaction and rarefaction for Shannon index (A), Richness of ARG subtypes (B) and Pielou's evenness (C).

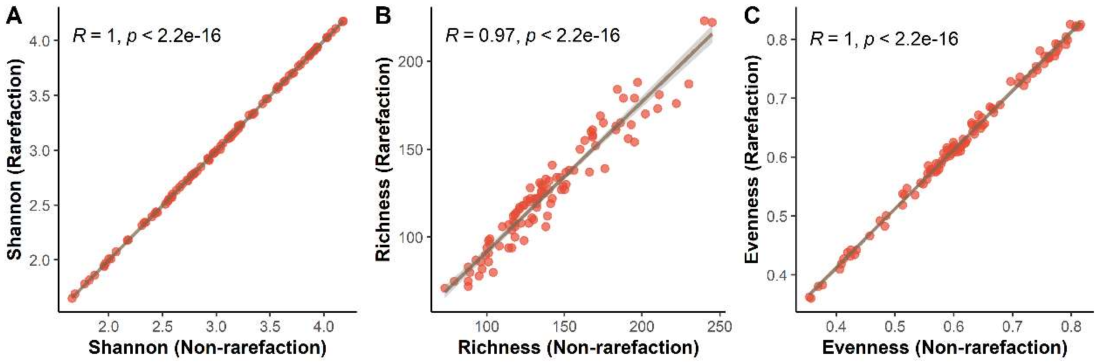

Fig. S4. Abundance and prevalence of gut antibiotic resistome in GNHS. A, The bar chart shows the prevalence of 17 antibiotic resistance genes (ARGs) types in participants with different diabetes status. **B**, The curve shows the association between ARGs subtype numbers and the prevalence among Healthy (n = 531), Prediabetes (n = 495) and T2D (n = 184) groups. **C**, The bar chart shows the prevalence of the ARGs types among different groups (differences between each two groups more than 3% are presented). **D**, The box plot shows the abundance of 17 core ARGs subtypes (prevalence = 100%). All box plots are the median with the interquartile range. *MLS*, *Macrolide-Lincosamide-Streptogramin*.

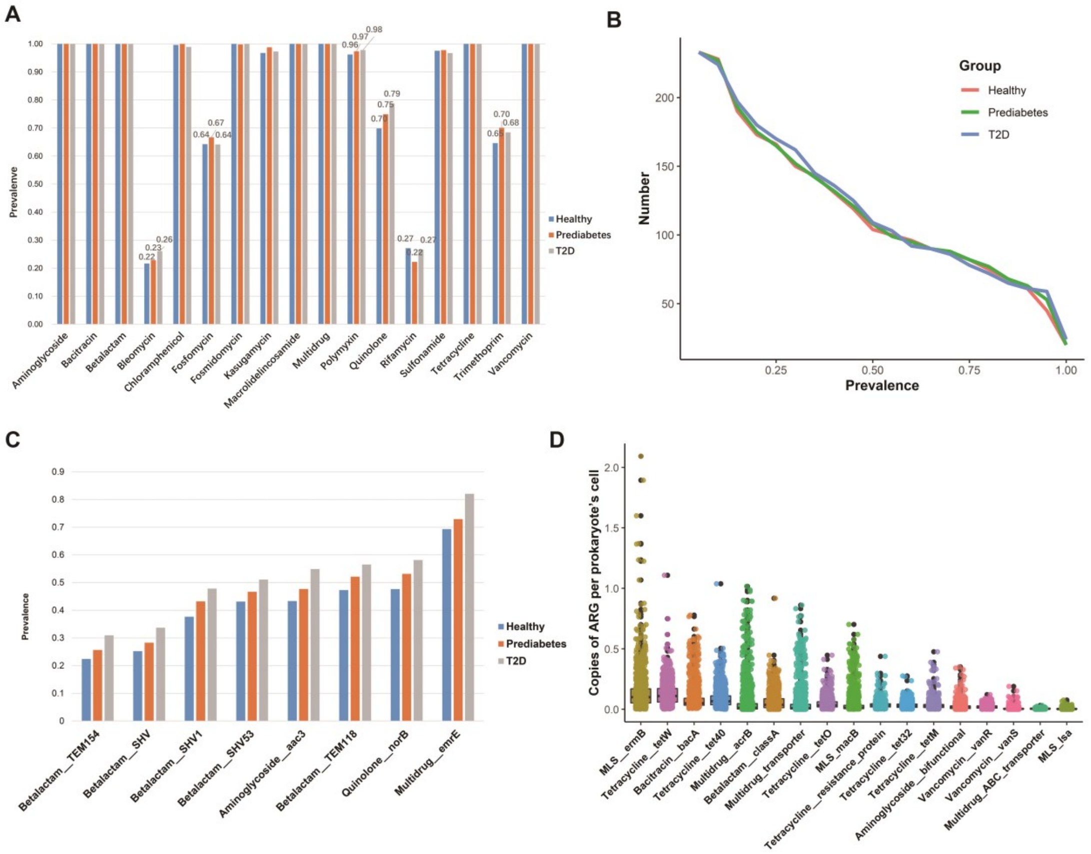

Fig. S5. The effect sizes of host factors in gut antibiotic resistome grouped by diabetes status. The effect sizes of host factors in human gut antibiotic resistome were calculated by PERMANOVA (Adonis, Bray-Curtis distance, permutations = 999) among Healthy (n = 392), Prediabetes (n = 401) and T2D (n = 154) groups.

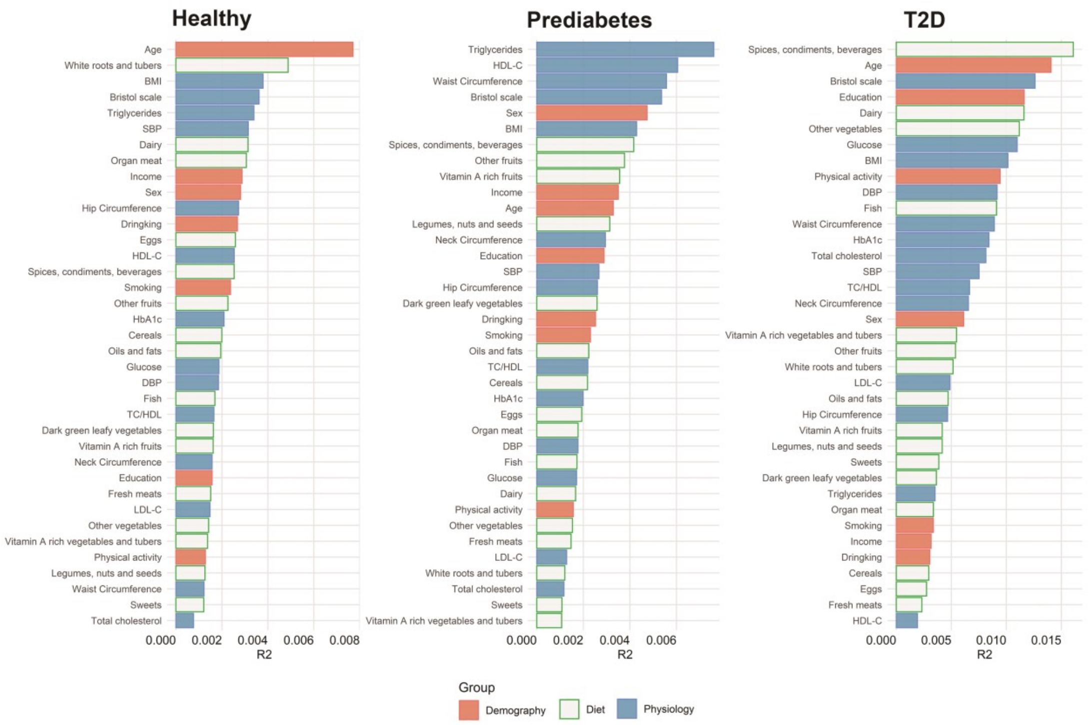

Fig. S6. Correlations between gut antibiotic resistome diversity and microbiota. **A**, **B,** Correlation between microbial gene richness (MGR) and α-diversity indices (ARGs) evaluated by Pearson tests. **C**, Procrustes analysis of gut ARGs versus gut microbiota. ARGs and microbiota are shown as orange and blue dots, respectively. ARGs and microbiota from the same individual are connected by grey lines.

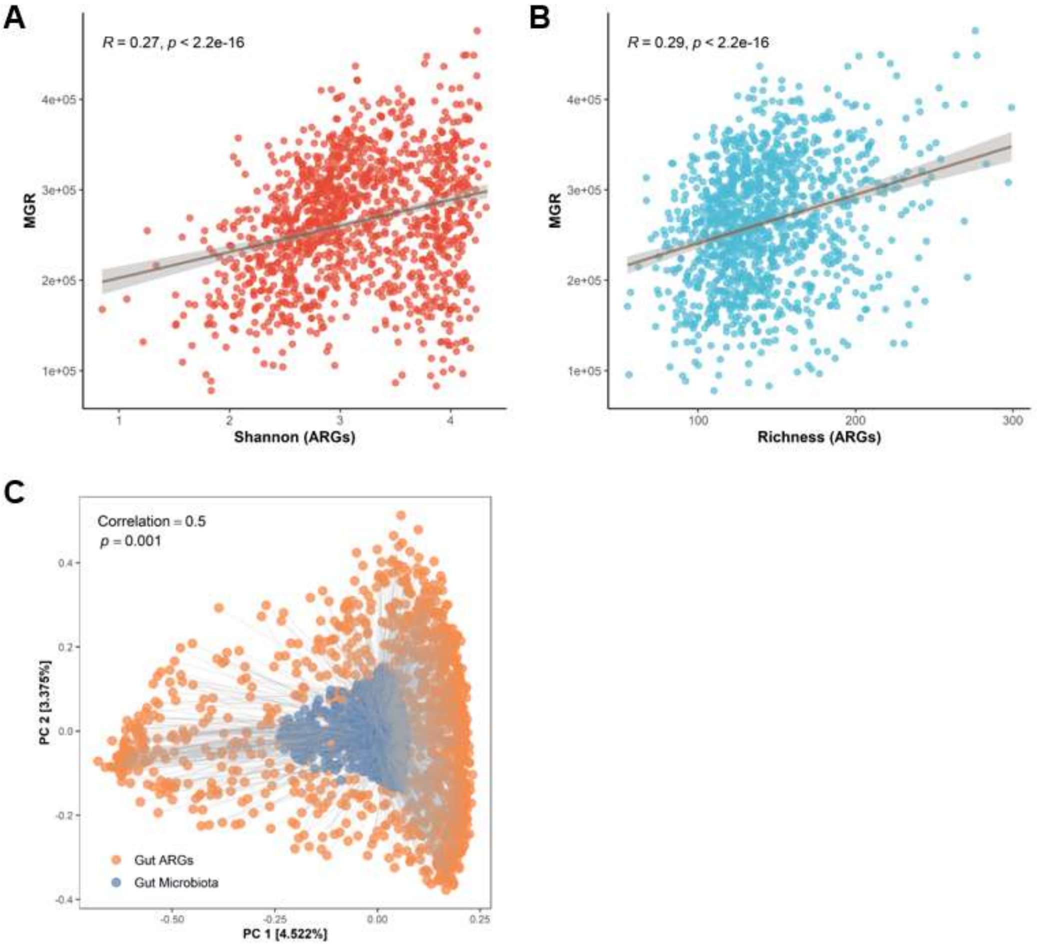

Fig. S7. The comparison of inter-individual Bray-Curtis distance of gut antibiotic resistome. Violin plots show the Bray-Curtis distance (y axis) among Healthy (n = 531), Prediabetes (n = 495) and T2D (n = 184) groups. *p* values from rank-based Wilcoxon test and Kruskal–Wallis test.

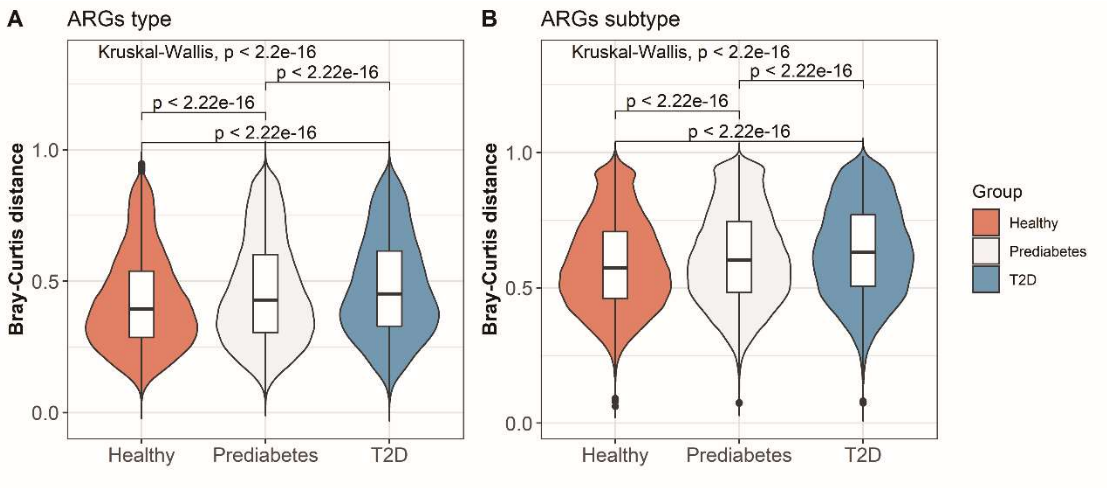

Fig. S8. The performance of models based on LASSO feature selection. LASSO regression models were performed with 5 repeated 5-fold cross-validations. The cross-validation AUCs were provided for both ARGs classifier (**A-C**) and microbiota classifier (**D-F**). Three dependent binary variables for antibiotic resistance genes marker selection: (**A**, **D**) Non-T2D (Healthy and Prediabetes)/T2D, (**B**, **E**) Healthy/T2D, (**C**, **F**) Prediabetes/T2D. LASSO, least absolute shrinkage and selection operator.

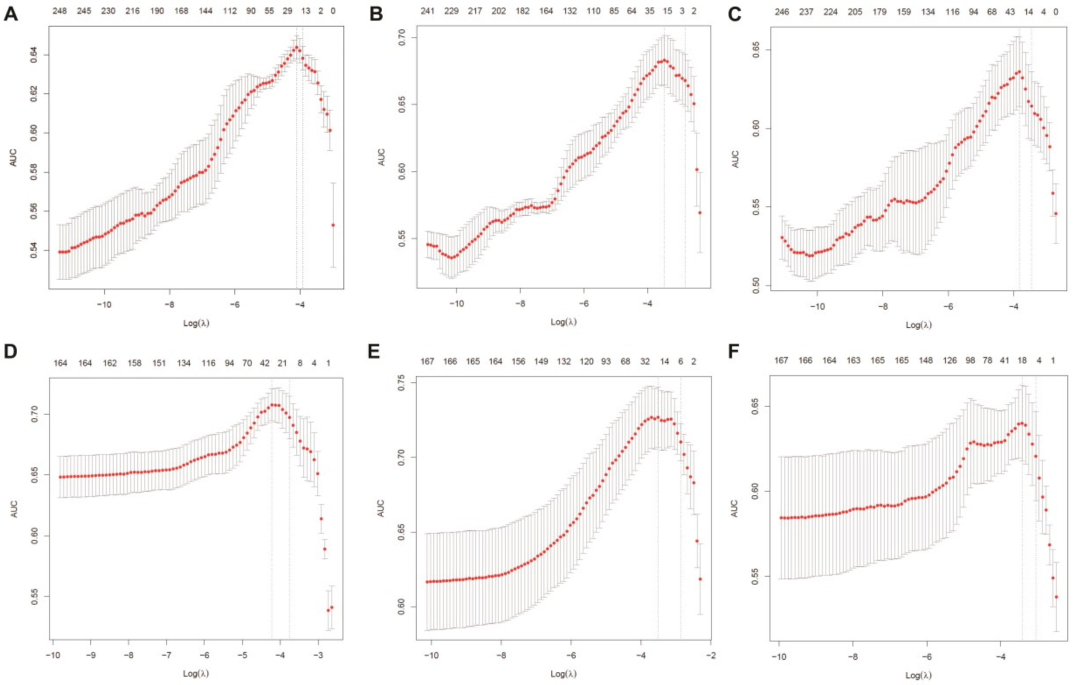

Fig. S9. Venn plot of biomarkers identified by LASSO models. Venn plot showing the number of biomarkers identified by gut ARGs classifier (**A**) and microbiota classifier (**B**) for different datasets: Non-T2D/T2D, Healthy/T2D and Prediabetes/T2D. LASSO, least absolute shrinkage and selection operator.

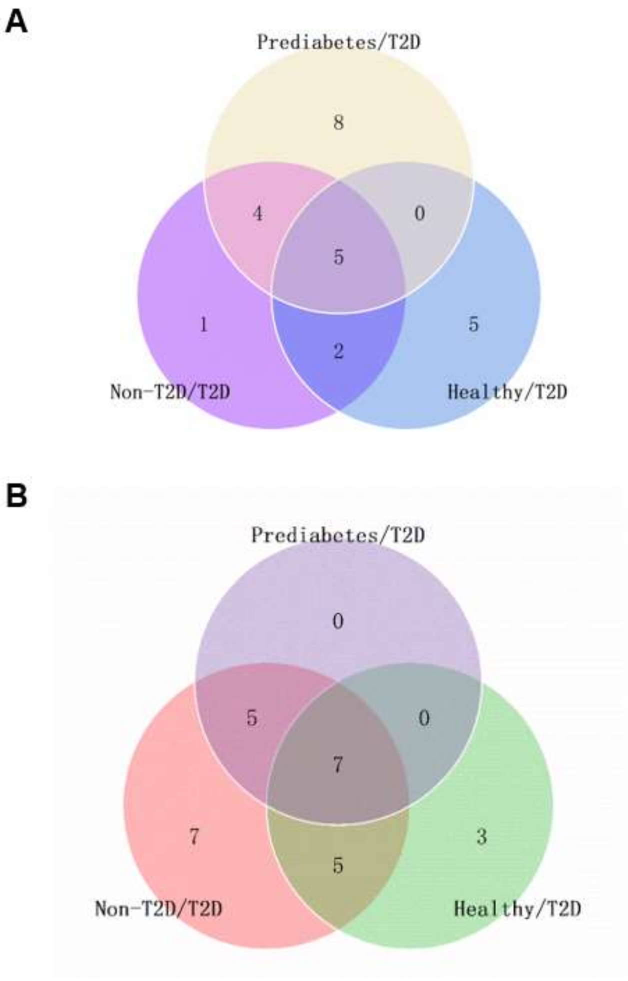

Fig. S10. Networks of co-occurring T2D-related ARGs and gut microbiota, grouped by diabetes status. Networks were presented based on correlation analysis among Healthy (n = 531), Prediabetes (n = 495) and T2D (n = 184) groups. A node stands for an ARG type/subtype or a species and a connection (i.e. edge) stands for a significant (FDR-corrected $p < 0.05$, Spearman's rho ≥ 0.3) pairwise correlation. Network was colored by ARGs and phylums. Node size is proportional to the number of connections (i.e. degree). *Ami*, *Aminoglycoside*; *Bet*, *Betalactam*; *Chl*, *Chloramphenicol*; *MLS*, *Macrolide-Lincosamide-Streptogramin*; *Mul*, *Multidrug*; *Qui*, *Quinolone*; *Tet*, *Tetracycline*; *Van*, *Vancomycin*.

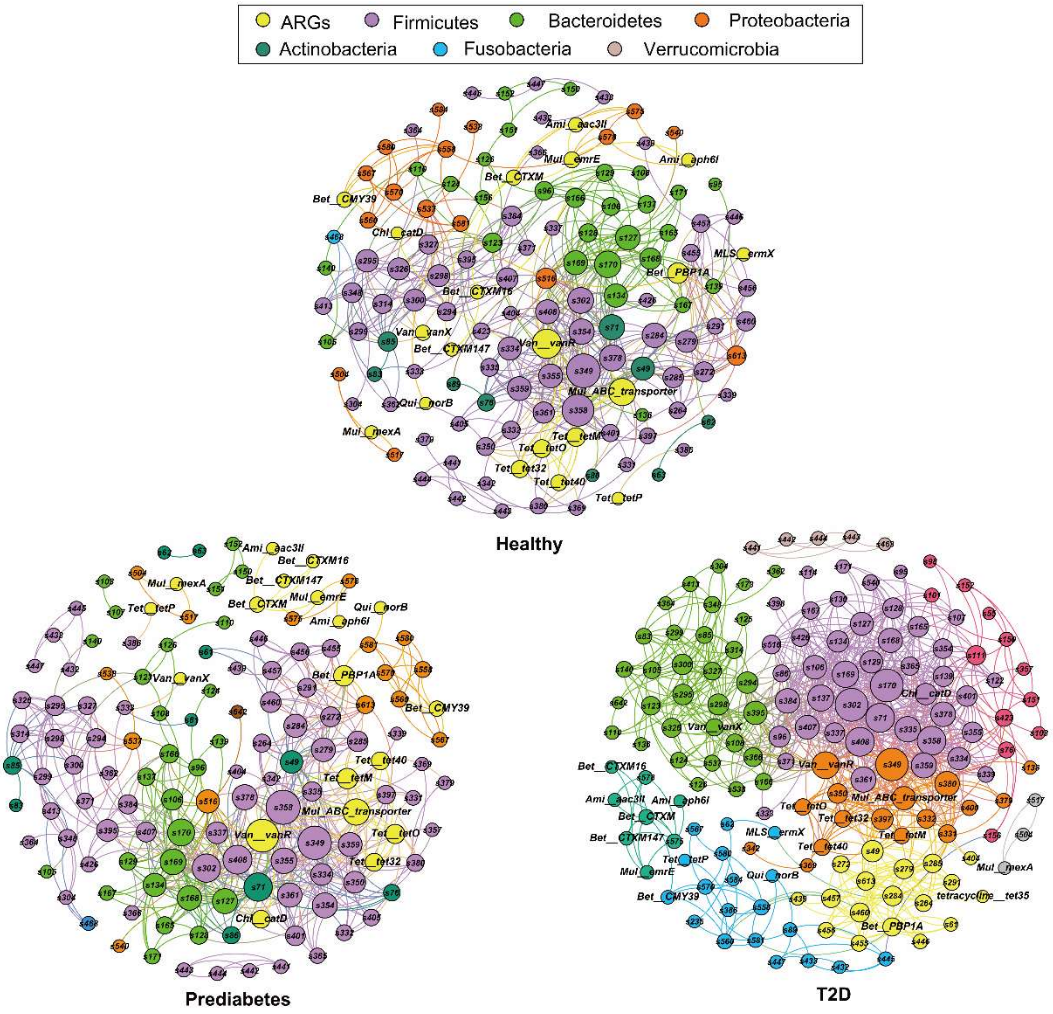

Fig. S11. Associations between gut antibiotic resistome features and fecal metabolites (n = 1012). The heatmap shows the Spearman correlation coefficients between gut antibiotic resistome features and fecal metabolites (purple text, showing fatty acids; yellow text, showing bile acids; blue text, showing amino acids). DAS, Diabetes-ARG score. *FDR-corrected p < 0.05, ** FDR-corrected p < 0.01, *** FDR-corrected p < 0.001.

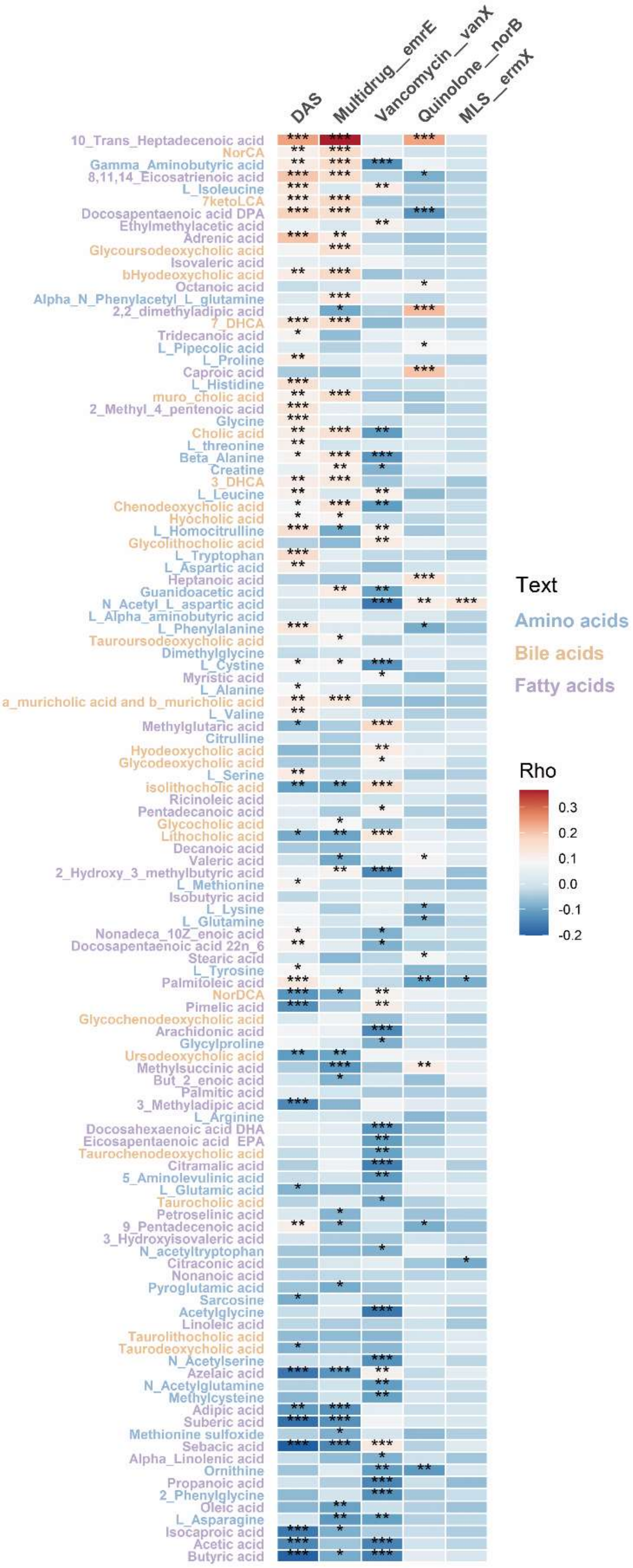

Supplement: Supplementary file 1 — Supporting Information [file ADVS-9-2104965-s001.pdf]
